# Supplementary material for: Search Algorithms as a Framework for the Optimization of Drug Combinations
Source: PLoS Comput Biol. 2008 Dec 26;4(12):e1000249. doi: 10.1371/journal.pcbi.1000249 (PMC2590660; doi:10.1371/journal.pcbi.1000249)
Supplement: Text S1 — Supplementary Material (0.42 MB DOC) [file pcbi.1000249.s001.doc]

**SUPPLEMENTARY MATERIAL**

Additional material and software is available at www.paternostrolab.org

**TABLE S1**

**
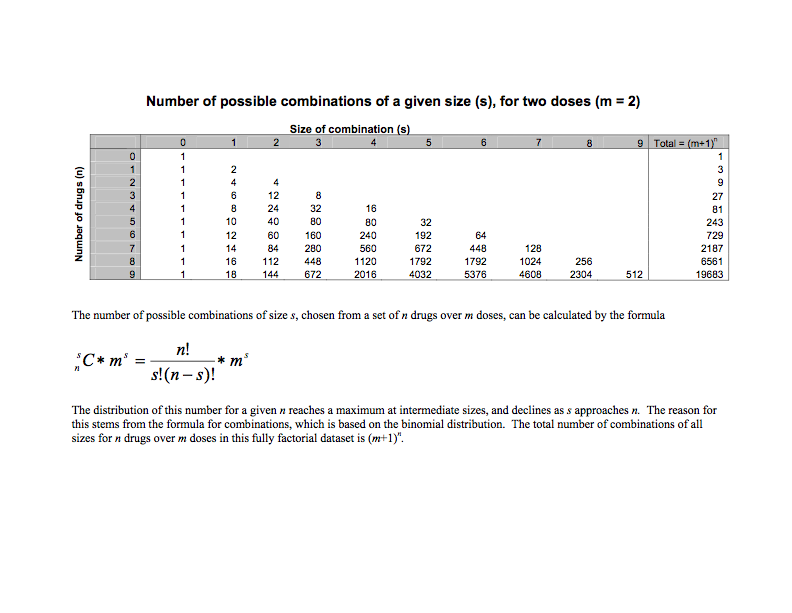
**

**TABLE S2**

**
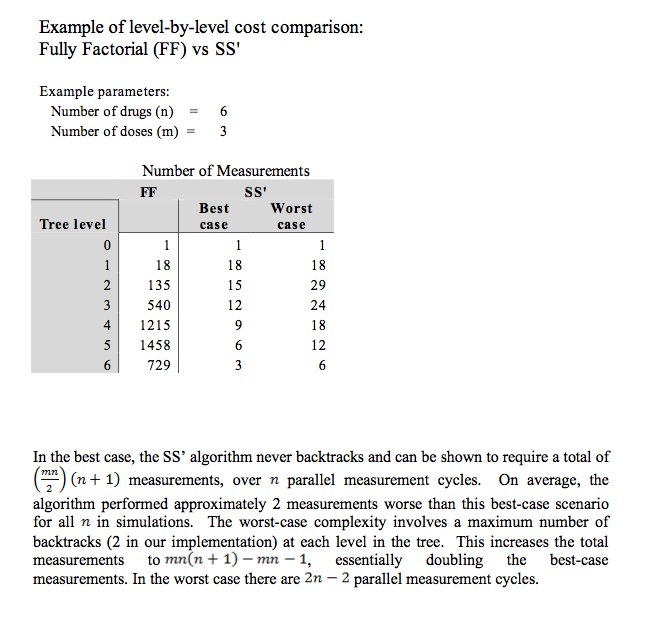
**


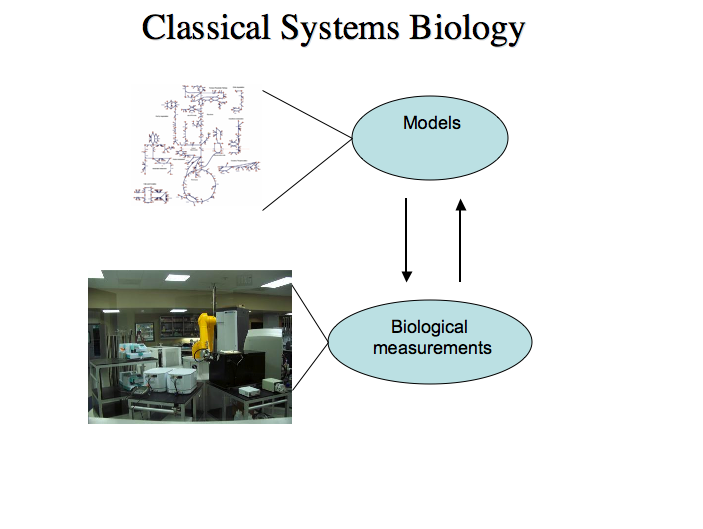


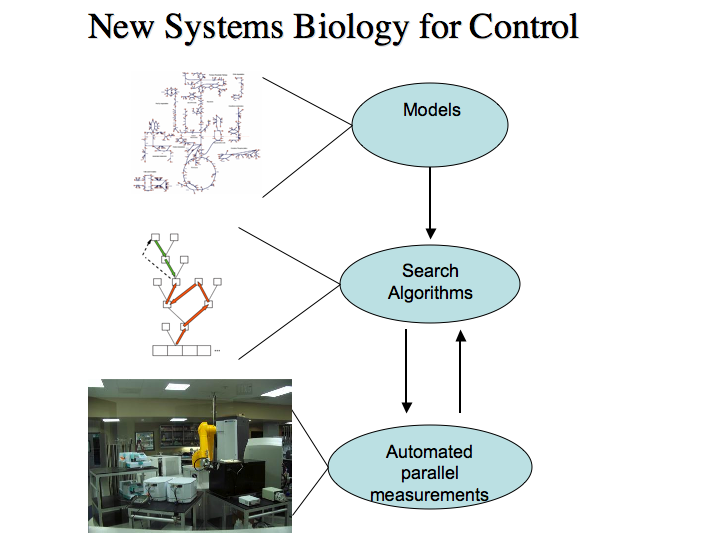


**FIGURE S1** – **Comparison of our approach with classical systems biology.**

The upper panel shows the classical approach to systems biology as a cycle of quantitative modeling and biological measurements. The lower panel shows the essential elements of the control-oriented approach we suggest. The middle element of the lower panel represents iterative algorithms that do not contain an explicit model of the reality to be controlled. The approach is supported by the appreciation of a fundamental limit of quantitative biological models (upper element of both panels), which are useful, indeed probably essential, but not sufficient for effective control. This limit is not only due to the complexity of biological organisms but also to their variation, which is one of the essential components of the process of natural selection, and therefore an unavoidable distinctive feature of biological systems.
